# Autonomous motivation to reduce sedentary behaviour is associated with less sedentary time and improved health outcomes in rheumatoid arthritis: a longitudinal study

**DOI:** 10.1186/s41927-022-00289-5

**Published:** 2022-10-10

**Authors:** Ciara M. O’Brien, Joan L. Duda, George D. Kitas, Jet J. C. S. Veldhuijzen van Zanten, George S. Metsios, Sally A. M. Fenton

**Affiliations:** 1 School of Psychology, University of Surrey, Guildford, UK; 2 School of Sport, Exercise and Rehabilitation Sciences, University of Birmingham, Edgbaston, Birmingham, B15 2TT UK; 3 Department of Rheumatology, Russells Hall Hospital, Dudley Group NHS Foundation Trust, West Midlands, UK; 4 Medical Research Council Versus Arthritis Centre for Musculoskeletal Ageing Research, University of Birmingham, Birmingham, UK; 5 Department of Nutrition and Dietetics, University of Thessaly, Thessaly, Greece

**Keywords:** Self-determination theory, Sedentary, Standing, Stepping, activPAL, Rheumatoid arthritis

## Abstract

**Background:**

This longitudinal study investigated whether changes in autonomous and controlled motivation to reduce sedentary behaviour were associated with variability in sedentary, standing and stepping time and, in turn, disease activity, systemic inflammation, pain and fatigue in rheumatoid arthritis (RA).

**Methods:**

People with RA undertook assessments at baseline (T1, *n* = 104) and 6 months follow-up (T2, *n* = 54) to determine autonomous and controlled motivation to reduce sedentary behaviour (Behavioural Regulation in Exercise Questionnaire-2), free-living sedentary, standing and stepping time (7 days activPAL3^μ^ wear), Disease Activity Score-28 (DAS-28), systemic inflammation (c-reactive protein [CRP]), pain (McGill Pain Questionnaire) and fatigue (Multidimensional Assessment of Fatigue Scale). *N* = 52 participants provided complete data at T1 and T2. *Statistical analyses:* In a series of models (A and B), path analyses examined sequential associations between autonomous and controlled motivation to reduce sedentary behaviour with activPAL3^μ^-assessed behaviours and, in turn, RA outcomes.

**Results:**

Models demonstrated good fit to the data. *Model A *(*sedentary and stepping time*)*:* autonomous motivation was significantly negatively associated with sedentary time and significantly positively related to stepping time. In turn, sedentary time was significantly positively associated with CRP and pain. Stepping time was not significantly associated with any health outcomes. *Model B *(*standing time*)*:* autonomous motivation was significantly positively associated with standing time. In turn, standing time was significantly negatively related to CRP, pain and fatigue.

**Conclusions:**

Autonomous motivation to reduce sedentary behaviour is associated with sedentary and standing time in RA which may, in turn, hold implications for health outcomes.

## Introduction

People living with rheumatoid arthritis (RA) have chronically elevated systemic inflammation [1]. To manage RA, clinicians adopt “treat-to-target” pharmacological approaches (e.g., disease-modifying anti-rheumatic drugs), with the aim of stringently controlling inflammatory disease activity [2]. However, despite pharmacological intervention, some individuals continue to frequently experience disease flares (increased levels of disease activity and inflammatory biomarkers) and report high levels of pain and fatigue [3]. Non-pharmacological, self-management approaches are now being increasingly used as an adjunct to pharmacological intervention to gain tighter control over RA disease activity. For example, strong evidence indicates that engagement in physical activity (PA) of a moderate-to-vigorous intensity (MVPA ≥ 3 metabolic equivalents [METs]) improves RA outcomes, such as disease activity, functional disability, pain and fatigue [4–8]. However, MVPA is a challenge for people with RA, which may be a reason for low levels of engagement in this population [9].

Movement exists on a continuum, ranging from sedentary behaviour (any waking behaviour expending energy ≤ 1.5 METs whilst sitting, reclining or lying) [10], to light-intensity PA (LPA 1.6–2.9 METs), to MVPA. Research indicates that people with RA spend most of the day engaged in sedentary behaviour (~ 60%) or LPA (~ 35%, e.g., standing, slow walking) [9] and that these high levels of sedentary time may exacerbate disease outcomes in RA (e.g., disease activity, functional disability and pain) [9, 11, 12]. Thus, whilst MVPA is most often advocated by health professionals to assist in self-management of RA [13], the health impacts of reducing sedentary time and increasing LPA (i.e., moving more) should not be discounted.

When we consider the strong inverse correlation between sedentary time and LPA (i.e., as sedentary time decreases throughout the day, time spent in LPA increases, and vice versa), it holds that increasing engagement in LPA (such as standing) may be a feasible and effective approach towards targeting reductions in sedentary time in RA. With this in mind, it is critical to not only elucidate the associations between sedentary time and LPA with pertinent RA outcomes, but to also identify modifiable determinants of both sedentary time and LPA which can be targeted via behaviour change interventions.

Motivation as a determinant of sedentary behaviour has been ranked as a research priority in The Systems of Sedentary behaviours (SOS-framework) [14]. Self-determination theory (SDT) [15] provides a relevant psychological framework to understand the motivational processes underlying health behaviour change and has been effectively applied in a PA context [16]. A central tenet of SDT is the concept of “quality of motivation”, proposed to lie on a continuum from controlled motivation (lower quality) to autonomous motivation (higher quality) [15, 17]. Autonomous motivation is reported to be operating where behaviour is directed by intrinsic motivation (e.g., enjoyment) and/or identified regulation (e.g., valuing the benefits), resulting in more optimal engagement and maintenance of the targeted behaviour. Controlled motivation, specifically introjected regulation (e.g., engaging in behaviour to avoid feelings of guilt) and/or external regulation (e.g., external pressure), is proposed to hold negative implications for uptake of and adherence to the targeted behaviour [17].

SDT has been used to understand the determinants of PA, including both MVPA and LPA in RA. For example, Hurkmans et al. [18] revealed that higher autonomous motivation was significantly associated with more self-reported PA in RA. More recently, Fenton et al. [19] demonstrated that receiving a 3 months SDT-based PA intervention was related to higher autonomous motivation and in turn, self-reported MVPA in RA. No studies, however, have investigated how the psychological processes outlined by SDT may be relevant to sedentary time in this population. That is, to what extent does an individual’s degree of autonomous and controlled motivation to reduce sedentary behaviour relate to changes in this behaviour and, in turn, improved health outcomes in RA.

It is important to examine the relationships between the modifiable determinants and pertinent health outcomes of sedentary behaviour and PA with levels of these movement behaviours (using valid measurement tools) in RA, to inform the design, delivery and evaluation of behaviour change interventions in this patient group [20]. The primary aim of this study was therefore to explore the degree by which changes in autonomous and controlled motivation to reduce sedentary behaviour were related to changes in device-measured sedentary time, standing time (an example of LPA) and stepping time (an indicator of total PA) and in turn, changes in clinically- and patient-important outcomes (disease activity, c-reactive protein [CRP], pain and fatigue) in people living with RA.

## Method

### Participants and recruitment

Individuals were approached in rheumatology outpatient clinics at Russells Hall Hospital in Dudley, England. Inclusion criteria were a clinical diagnosis of RA (American College of Rheumatology/European League Against Rheumatism Classification Criteria) [21] and an age of ≥ 18 years. Individuals were excluded if they were pregnant, wheelchair users and/or unable to ambulate independently with the use of an assistive device. Willing patients provided informed consent to participate. This study was granted ethical approval by the West Midlands National Health Service Research Ethics Committee (16/WM/0371).

### Protocol

The protocol for this longitudinal study has been published elsewhere [22]. In brief, participants were asked to visit the hospital at baseline (Time 1 [T1]) and 6 months follow-up (Time 2 [T2]). T1 and T2 comprised two visits each, separated by 7-days (visit 1 [day 0] and visit 2 [day 7]). During visits, participants completed routine clinical procedures, physical assessments and questionnaires, and were fitted with an activPAL3^μ^ (PAL Technologies Ltd., Glasgow, UK) to wear for the subsequent 7 days (to assess sedentary, standing and stepping time). Data was collected between February 2017 and June 2018.

### Measures

#### Visit 1 (day 0)

##### Demographic information, medical history and physical assessments

Participants’ sex, age, ethnicity, marital status, date of diagnosis, existing chronic conditions and treatment regime were recorded. Then, height (cm), weight (kg) body-mass index (kg/m^2^) and resting blood pressure (mmHg) were measured in duplicate by the same researcher for each participant.

##### Health assessment questionnaire

The Health Assessment Questionnaire (HAQ) is routinely used to assess physical function in clinical practice [23]. The HAQ assesses an individual’s ability to carry out 8 activities of daily living (ADLs [e.g., walking, reach, grip]). Participants were asked to self-report their ability to undertake specific tasks associated with each ADL over the previous 2 weeks, on a 4-point rating scale (0 = without any difficulty; 1 = with some difficulty; 2 = with much difficulty; 3 = unable to do). Average HAQ scores were computed (higher scores indicated poorer physical function: min score = 0; max score = 3). In this study, the HAQ showed high internal reliability (α = 0.91).

##### Behavioural regulation in exercise questionnaire-2 (adapted for reducing sedentary behaviour)

The Behavioural Regulation in Exercise Questionnaire-2 (BREQ-2) [15, 24] measures an individual’s degree of autonomous and controlled motivation towards exercise. The BREQ-2 has been adapted to examine the associations between quality of motivation to engage in PA with levels of PA participation in RA [25]. In this study, the BREQ-2 was modified to measure autonomous and controlled motivation to reduce sedentary behaviour. Specifically, the stem, “I take part in exercise, because” was changed to, “I aim to reduce my sedentary behaviour, because”. Participants were asked to respond to items relating to intrinsic regulation (4 items; e.g., “I enjoy doing this”), identified regulation (4 items; e.g., “I value the benefits of doing this”), introjected regulation (3 items; e.g., “I feel guilty when I am not doing this”) and external regulation (4 items; e.g., “my friends and family say I should”). Participants rated their agreement with each statement on a 5-point scale (1 = strongly disagree; 2 = disagree; 3 = neutral; 4 = agree; 5 = strongly agree) referring to the previous 4 weeks. Previous research in adolescents has adapted the BREQ-2 in this way to assess quality of motivation to reduce screen time (a specific sedentary behaviour) [26].

The adapted BREQ-2 employed in this study started with a definition of “reducing sedentary behaviour” to help participants understand its meaning (“reducing sedentary behaviour refers to your overall attempts to spend less time sitting or lying down, *not* just your attempts to more frequently interrupt periods of sitting with physical activity or standing”). Before completing the questionnaire, the researcher checked the participant’s understanding of what was meant by “reducing sedentary behaviour” as conceptualised in this study, using standardised language.

Average scores were computed for each regulation and summed to generate composite scores for autonomous motivation (intrinsic regulation + identified regulation) and controlled motivation (introjected regulation + external regulation). Higher scores for autonomous and controlled motivation indicated higher levels of these regulations (min score = 2; max score = 10). The adapted BREQ-2 revealed high internal reliability for autonomous (α = 0.87) and controlled motivation (α = 0.81) to reduce sedentary behaviour in this study.

#### Sedentary, standing and stepping time

##### activPAL3^μ^

The activPAL3^μ^ is an accelerometer that measures free-living sedentary, standing and stepping time over continuous 24-h periods. This device is considered the gold standard measure of free-living sedentary time [27] and has recently been validated for the measurement of sedentary, standing and stepping time in people living with RA [28]. In this study, PAL Connect software (PAL Technologies Ltd., Glasgow, UK) was used to initialise the activPAL3^μ^ to record free-living behaviour in 15-s epochs. The device was fitted by the researcher, attached to the mid-anterior position of the participant’s right thigh with an adhesive, waterproof dressing [29]. Participants were asked to wear the activPAL3^μ^ for 24 h/day for the 7 days between visits 1 and 2, and to record any removal of the device in a wear time logbook.

Using PAL Connect, participants’ activPAL3^μ^ data were downloaded and exported to Microsoft Excel. The researcher manually removed sleep time from sedentary time estimates, and this was double-checked by another member of the research team. Wear time criteria for inclusion in statistical analyses were: activPAL3^μ^ wear for ≥ 10 h/day on ≥ 4 days, including ≥ 1 weekend day [29]. Participants must have met these wear time criteria at both T1 and T2 to be included in the longitudinal analyses. For participants with valid activPAL3^μ^ data, average daily waking time spent sedentary, standing and stepping (min/day), as well as the average daily percentage (%) of waking time spent in these behaviours, were calculated for use in statistical analyses (e.g., activPAL3^μ^-assessed sedentary time per day [%] = (activPAL3^μ^-assessed sedentary time [min/day]/total activPAL3^μ^ wear time [min/day]) × 100).

#### Visit 2 (day 7)

##### Fasting blood sample

After a ≥ 12-h fast, participants provided a blood sample to measure serum biomarkers of inflammation. Specifically, erythrocyte sedimentation rate (ESR [mm/h]) and serum CRP (mg/l) were measured using standard laboratory procedures and Enzyme-Linked Immunosorbent Assays (MP Biomedicals, UK), respectively.

##### Disease activity score-28

The Disease Activity Score-28 (DAS-28) was employed as a measure of RA disease activity in this study. DAS-28 is a validated measure of RA disease activity that is used routinely in clinical practice [30, 31] and is determined using a clinical calculator. Data entered into the calculator are as follows; the number of swollen and tender synovial joints (clinician/researcher reported and patient confirmed, from 28 joints), degree of overall self-rated health (visual analogue scale from 0 = very good to 100 = very poor) and ESR. Clinical interpretation of DAS-28 scores: ≤ 3.2 = low disease activity; > 3.2 − ≤ 5.1 = moderate disease activity; > 5.1 = high disease activity [32].

##### McGill pain questionnaire

The McGill Pain Questionnaire (MPQ) assesses multidimensional aspects of pain in RA and has been validated for use in this patient group [33, 34]. This 15-item questionnaire captures both sensory (11 items, e.g., “hot-burning”) and affective (4 items, e.g., “cruel-punishing”) dimensions of pain. Participants rated the degree by which they experienced each item over the previous 7 days on a 4-point scale (0 = none; 1 = mild; 2 = moderate; 3 = severe). Responses were summed to compute a total pain score (higher scores = higher pain: min score = 0; max score = 45). In this study, the MPQ showed high internal reliability (α = 0.93).

##### Multidimensional assessment of fatigue scale

The Multidimensional Assessment of Fatigue Scale (MAF) has been developed, validated and extensively employed to measure global fatigue in RA [35–37]. The 15-item MAF required participants to consider the previous 7 days and indicate their degree of fatigue (rating scale from 1 = not at all to 10 = a great deal), severity of fatigue (rating scale from 1 = mild to 10 = severe), and to what extent fatigue caused them distress (rating scale from 1 = no distress to 10 = a great deal of distress) and interfered with the ability to carry out ADLs (e.g., “bathe or wash” [rating scale from 1 = not at all to 10 = a great deal]), over the previous 7 days. A global fatigue index was calculated (higher scores = higher fatigue: min score = 0; max score = 50). The MAF demonstrated high internal reliability in this study (α = 0.98).

### Statistical analyses

Statistical analyses were conducted using SPSS and AMOS software (version 24). Descriptive statistics were computed for variables at T1 and T2. Change in variables from T1 to T2 were calculated (change score = T2–T1) for use in longitudinal analyses. Kolmogorov–Smirnov tests of normality and visual inspection of graphs (histograms, Q–Q plots) established that data at T1 and the change scores were not entirely normally distributed, therefore, bias-corrected and accelerated bootstrapping (a non-parametric resampling procedure) was employed in subsequent analyses. Bootstrapping involves intensively resampling data (typically x ≥ 1000 samples) from the original sample data to establish 95% confidence intervals (CIs), which are interpreted to determine statistical significance [38–40]. Bias-corrected and accelerated bootstrapping is advocated to deal with non-normal data in small sample sizes [40–43].

Bivariate Pearson’s correlation analyses examined cross-sectional and longitudinal associations between autonomous and controlled motivation to reduce sedentary behaviour, sedentary, standing and stepping time (% waking behaviour per day, to adjust for variability in activPAL3^μ^ wear time) and the targeted RA outcomes (DAS-28, CRP, pain and fatigue). Relationships between variables were classed as significant if bootstrapped 95% CIs did not cross zero. Standardised non-bootstrapped coefficients (*β*) indicated the strength of associations (small = 0.10; moderate = 0.30; large = 0.50) [44].

**Fig. 1:**
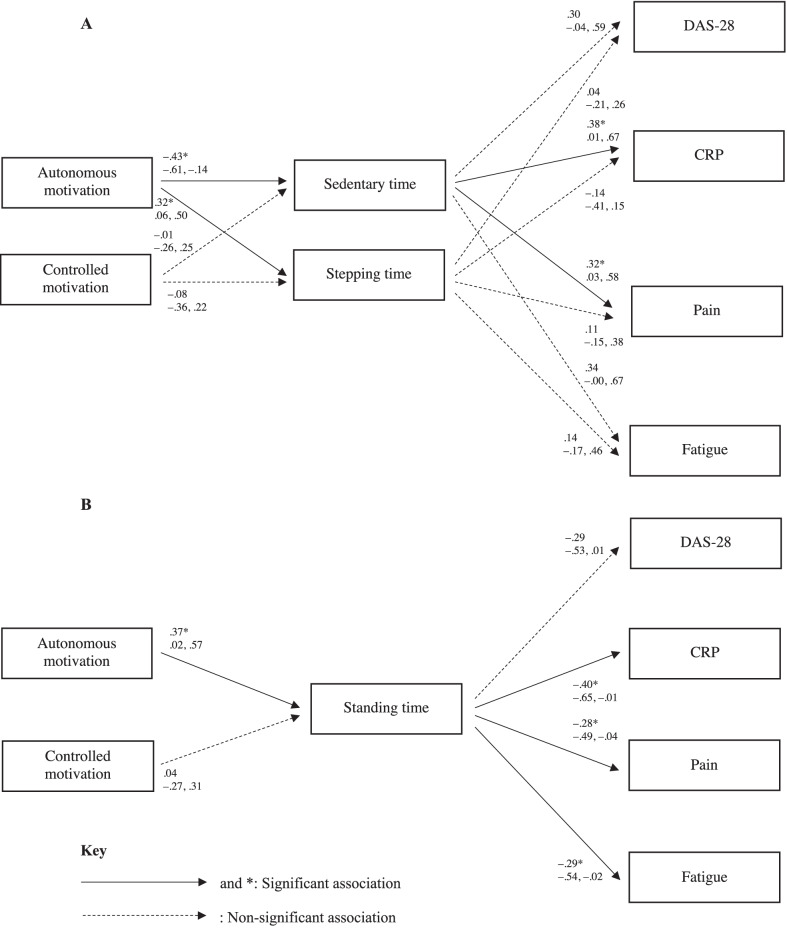
Path analyses models: Model** A** (sedentary and stepping time)—sequential associations between change in autonomous and controlled motivation to reduce sedentary behaviour with change in sedentary and stepping time and, in turn, change in Disease Activity Score-28 (DAS-28), c-reactive protein (CRP), pain and fatigue in people with RA; Model** B** (standing time)—sequential associations between change in autonomous and controlled motivation to reduce sedentary behaviour with change in standing time and, in turn, change in DAS-28, CRP, pain and fatigue in people with RA

Longitudinal data, rather than cross-sectional data, was then used in path analyses to test two hypothesised models, examining sequential relationships between change in autonomous and controlled motivation to reduce sedentary behaviour with change in activPAL3^μ^-assessed sedentary time, standing time and stepping time and, in turn, change in DAS-28, CRP, pain and fatigue. Indeed, cross-sectional data only provides a “snapshot” of information (collected at a single point in time), whilst longitudinal data can offer important insight into how change in one variable (e.g., sedentary time) relates to change in another (e.g., RA-related pain) over time. The hypothesised models were constructed as depicted in Fig. 1, and according to the following rationale.

First, autonomous and controlled motivation to reduce sedentary behaviour were concurrently incorporated into the models as exogenous variables. Controlled motivation is an important construct within SDT, and it is possible for an individual to be autonomously motivated and holding controlled motivation towards a specific behaviour [45]. For example, an individual may experience both autonomous (e.g., because they value the benefits) and controlled (e.g., because someone tells them to) motivation to reduce sedentary behaviour at the same time. Thus, it is important to understand the independent and relative contributions of each.

Second, sedentary time and stepping time (an indicator of total PA) were included simultaneously in models as endogenous variables (sequentially predicted by autonomous and controlled motivation) to assess their independent and relative effects on RA outcomes. A high correlation between sedentary and standing time (*β* = − 0.95), indicating multicollinearity between these variables, resulted in the need to examine standing time in a separate model to sedentary and stepping time. The implications of time spent standing for RA outcomes is as important to examine as both sedentary and stepping time, as it presents a lower-intensity activity (LPA) that could be perceived as a more feasible alternative to MVPA for people with RA.

Finally, DAS-28, CRP, pain and fatigue were modelled together to represent “clinically-important” (DAS-28 and CRP) and “patient-important” (pain and fatigue) outcomes (according to the Outcome Measures in Rheumatology initiative) [46].

To ensure adequate statistical power, all variables were modelled as observed variables to reduce the number of parameters in the model. Specifically, due to the small sample size, we were not able to test a full measurement model (specifying latent variables) for factors assessed via questionnaire.

Path analyses with maximum likelihood estimation, in conjunction with bias-corrected and accelerated bootstrapping (1000 samples), was used to test all models. Statistically significant direct and indirect relationships between variables were determined by examination of bootstrapped 95% CIs, and standardised coefficients (*β)* facilitated interpretation of the strength of each association. Model fit was evaluated via examining the chi-square statistic (χ^2^), comparative fit index (CFI), Tucker Lewis index (TLI) and root mean square error of approximation (RMSEA; 90% CIs). A non-significant χ^2^ (*p* > 0.05), CFI and TLI values ≥ 0.90, and RMSEA < 0.08 with 90% CIs (lower boundary) < 0.05 suggests good model fit [47, 48].

## Results

At T1, *n* = 104 participants undertook assessments, with *n* = 102 participants providing complete data (non-compliance with the activPAL3^μ^ for *n* = 2 participants). Of those recruited at T1, *n* = 54 participants (52%) completed T2 assessments (participants lost at T2 were due to time and funding constraints). Data from *n* = 52 of these participants were available for longitudinal analyses (activPAL3^μ^ malfunction plus no CRP data available for *n* = 1 participant, and no CRP data available for an additional *n* = 1 participant) (Table 1).

**Table 1:** Descriptive statistics for sample at T1 and T2, and change from T1 to T2

	*n*	T1	*n*	T2	*n*	Change
Age (years)	102	58.3 (12.3)	53	58.9 (12.2)	–	–
Sex (% female)	72	71	37	70	–	–
Ethnicity (% Caucasian)	97	95	51	96	–	–
Marital status (% married)	66	65	38	70	–	–
*RA disease*						
RA duration (years)	102	10.4 (10.5)	53	9.0 (8.1)	–	–
Physical function (HAQ)	102	1.2 (0.8)	53	1.0 (0.8)	–	–
DMARDs (% on DMARDs)	92	90	46	87	–	–
Anti-TNF (% on anti-TNF)	15	14	11	20	–	–
NSAIDs (% on NSAIDs)	19	18	11	20	–	–
*Physical health*						
Height (m)	102	1.7 (0.1)	53	1.7 (0.1)	–	–
Weight (kg)	102	80.0 (20.3)	53	81.7 (22.0)	–	–
BMI (kg/m^2^)	102	29.1 (6.1)	53	29.7 (6.6)	–	–
Systolic BP (mmHg)	102	129 (15)	53	132 (13)	–	–
Diastolic BP (mmHg)	102	77 (9)	53	77 (8)	–	–
*Quality of motivation*						
Autonomous motivation (BREQ-2)	102	7.2 (1.4)	53	7.5 (1.5)	53	− 0.1 (1.2)
Controlled motivation (BREQ-2)	102	4.3 (1.6)	53	4.3 (1.7)	53	− 0.0 (1.7)
*activPAL3*^*μ*^*™ data*						
Valid wear time (min/day)	102	913.0 (56.7)	53	941.3 (60.4)	53	20.5 (54.2)
Sedentary time (min/day)	102	546.1 (116.6)	53	574.8 (98.8)	53	37.9 (65.3)
Standing time (min/day)	102	267.5 (101.0)	53	266.6 (92.7)	53	− 13.1 (59.9)
Stepping time (min/day)	102	99.4 (37.4)	53	99.9 (40.3)	53	− 4.3 (19.8)
Sedentary time (%/day)	102	60.0 (12.9)	53	61.4 (11.6)	53	2.8 (6.8)
Standing time (%/day)	102	29.2 (10.5)	53	28.1 (8.9)	53	− 2.1 (5.9)
Stepping time (%/day)	102	10.9 (4.0)	53	10.5 (4.0)	53	− 0.7 (2.2)
*Clinically-important outcomes*						
DAS-28	102	4.0 (1.5)	53	4.0 (1.5)	53	0.2 (1.3)
CRP (mg/l)	102	6.1 (7.6)	52	6.2 (8.2)	52	0.9 (8.3)
*Patient-important outcomes*						
Pain (MPQ)	102	12.8 (11.0)	53	13.4 (11.0)	53	− 0.7 (10.0)
Fatigue (MAF)	102	24.8 (13.2)	53	23.6 (13.2)	53	− 1.5 (8.7)

^a^Values are percentages (%) and mean (standard deviation)

^b^*n* = number of participants; T1 = time 1; T2 = time 2; RA = rheumatoid arthritis; HAQ = Health Assessment Questionnaire; DMARDs = disease-modifying anti-rheumatic drugs; Anti-TNF = anti-tumour necrosis factor; NSAIDs = non-steroidal anti-inflammatory drugs; BMI = body-mass index; BP = blood pressure; BREQ-2 = Behavioural Regulation in Exercise Questionnaire-2; DAS-28 = Disease Activity Score-28; CRP = c-reactive protein; MPQ = McGill Pain Questionnaire; MAF = Multidimensional Assessment of Fatigue Scale

Independent samples *t*-test and chi-square analyses demonstrated no significant differences between participants included at both T1 and T2 (*n* = 54) and those lost between time points (*n* = 50) regarding all measured variables (all *p* > 0.05), with the exception of pain (*p* < 0.05). The difference in mean values for pain between participants included at both at T1 and T2 and those lost between time points was small (3.0), and both mean values were within the same “range” of pain on the MPQ (i.e., mild pain).

### Bivariate correlation analyses

Table 2 shows the results from bootstrapped cross-sectional (T1) and longitudinal (change from T1 to T2) bivariate correlation analyses. Longitudinal analyses revealed that change in autonomous motivation to reduce sedentary behaviour was significantly negatively associated with change in sedentary time (*β* = − 0.43), and significantly positively related to change in standing (*β* = 0.38) and stepping (*β* = 0.33) time. Change in controlled motivation was not significantly linked to change in any activPAL3^μ^-assessed behaviours (sedentary time, *β* = 0.01; standing time, *β* = 0.03; stepping time, *β* = − 0.10). Significant positive relationships were shown between change in sedentary time with change in CRP (*β *= 0.45) and fatigue (*β* = 0.27), with the inverse demonstrated for change in standing time (CRP, *β* = − 0.40; fatigue, *β* = − 0.29). Change in standing time was also significantly negatively associated with change in pain (*β* = − 0.27). Change in sedentary time was not significantly linked to change in DAS-28 (*β* = 0.24) and pain (*β* = 0.26), change in standing time was not significantly related to change in DAS-28 (*β* = − 0.24), and change in stepping time was not significantly associated with change in any RA outcomes (DAS-28, *β* = − 0.11; CRP, *β* = − 0.34; pain, *β* = − 0.07; fatigue,* β* = − 0.05).

**Table 2:** Bivariate Pearson’s correlations between all variables (cross-sectional [T1] and longitudinal [change from T1 to T2] data)

	1	2	3	4	5	6	7	8
*T1*								
1 Autonomous motivation								
2 Controlled motivation	.05 (− .16 to .25)							
3 Sedentary time	− .28* (− .48 to − .07)	.16 (− .04 to .33)						
4 Standing time	.23* (.02 to .43)	− .12 (− .29 to .07)	− .96* (− .98 to − .95)					
5 Stepping time	.31* (.12 to .47)	− .19 (− .38 to .03)	− .71* (− .80 to − .62)	.50* (.35 to .64)				
6 DAS-28	− .08 (− .30 to .14)	.13 (− .08 to .32)	.29* (.11 to .45)	− .24*(− .39 to − .06)	− .31* (− .49 to − .12)			
7 CRP	− .09 (− .25 to .06)	− .11 (− .27 to .05)	.18 (− .04 to .38)	− .15 (− .34 to .04)	− .19* (− .34 to − .03)	.35* (.11 to .53)		
8 Pain	.07 (− .14 to .26)	.16 (− .04 to .36)	.21* (.03 to .38)	− .16 (− .33 to .01)	− .26* (− .42 to − .09)	.55* (.42 to .66)	.15 (− .05 to .35)	
9 Fatigue	− .16 (− .36 to .04)	.29* (.12 to .46)	.19* (.02 to .35)	− .15 (− .32 to .04)	− .23* (− .41 to − .01)	.53* (.38 to .67)	.21* (.05 to .36)	.72* (.62 to .80)
*Change (T1 to T2)*								
1 Autonomous motivation								
2 Controlled motivation	− .10 (− .32 to .17)							
3 Sedentary time	− .43* (− .61 to − .19)	.01 (− .27 to .30)						
4 Standing time	.38* (.10 to .58)	.03 (− .25 to .31)	− .95* (− .98 to − .91)					
5 Stepping time	.33* (.08 to .53)	− .10 (− .41 to .21)	− .55* (− .74 to − .28)	.27 (− .07 to .56)				
6 DAS-28	.02 (− .24 to .27)	− .07 (− .27 to .16	.24 (− .03 to .48)	− .24 (− .51 to .05)	− .11 (− .35 to .15)			
7 CRP	− .17* (− .31 to − .01)	− .20 (− .37 to .00)	.45* (.13 to .64)	− .40* (− .66 to − .01)	− .34 (− .56 to .01)	.60* (.35 to .75)		
8 Pain	− .00 (− .24 to .22)	− .05 (− .33 to .27)	.26 (− .00 to .47)	− .27* (− .50 to − .02)	− .07 (− .28 to .18)	.49* (.26 to .70)	.35* (.15 to .60)	
9 Fatigue	− .20 (− 39 to .02)	.10 (− .16 to .36)	.27* (.03 to .50)	− .29* (− .56 to − .01)	− .05 (− .32 to .20)	.31* (.09 to .49)	.28* (.04 to .53)	.57* (.29 to .76)

^a^Bias-corrected and accelerated bootstrapping was applied to bivariate Pearson’s correlation analyses to generate standardised beta coefficients (*β*) and 95% confidence intervals (lower to upper). Sedentary, standing and stepping time were calculated as percentages of activPAL3^μ^ wear time for use in bivariate Pearson’s correlations

^b^* = Significant association (95% confidence intervals derived from bootstrapping did not cross zero)

^c^T1 = time 1; T2 = time 2; DAS-28 = disease activity score-28; CRP = c-reactive protein

### Path analyses

Results from all models are illustrated in Fig. 1. All models demonstrated a good fit to the data (Model A: χ^2^ (11) = 13.53, *p* = 0.26, CFI = 0.97, TLI = 0.92, RMSEA = 0.07 [90% CI: 0.00 to 0.17]; Model B: χ^2^ (12) = 14.50, *p* = 0.27, CFI = 0.96, TLI = 0.93, RMSEA = 0.06 [90% CI: 0.00 to 0.16]). Standardised path coefficients (*β*) and 95% CIs (lower to upper) for direct associations are reported in the models (Fig. 1). This information for indirect associations is reported below.

Model A. *Direct effects:* Change in autonomous motivation to reduce sedentary behaviour was significantly negatively associated with change in sedentary time, and significantly positively related to change in stepping time. In turn, change in sedentary time was positively linked to change in CRP and pain. No significant associations were shown between; (1) change in controlled motivation to reduce sedentary behaviour with change in sedentary time and stepping time, (2) change in sedentary time with change in DAS-28 and fatigue, and (3) change in stepping time with change in DAS-28, CRP, pain and fatigue. *Indirect effects:* A significant negative indirect association was demonstrated between change in autonomous motivation to reduce sedentary behaviour with change in CRP (*β* = − 0.21, 95% CIs = − 0.37 to − 0.04), via change in sedentary and stepping time.

Model B. *Direct effects:* Change in autonomous motivation to reduce sedentary behaviour was significantly positively related to change in standing time and, in turn, negatively associated with change in CRP, pain and fatigue. No significant associations were shown between; (1) change in controlled motivation to reduce sedentary behaviour with change in standing time, and (2) change in standing time with change in DAS-28. *Indirect effects:* A significant negative indirect association was shown between change in autonomous motivation to reduce sedentary behaviour with change in CRP (*β* = − 0.15, 95% CIs = − 0.33 to − 0.00), pain (*β* = − 0.10, 95% CIs = − 0.22 to − 0.01) and fatigue (*β* = − 0.11, 95% CIs = − 0.25 to − 0.00), via change in standing time.

## Discussion

This is the first study to examine the sequential relationships between autonomous and controlled motivation to reduce sedentary behaviour with sedentary, standing and stepping time and, in turn, clinically- and patient-important outcomes in RA. Path analyses revealed that change in autonomous motivation to reduce sedentary behaviour was consistently negatively associated with change in sedentary time. In turn, change in sedentary time was positively associated with change in CRP and pain. Therefore, autonomous motivation to reduce sedentary behaviour may represent an important modifiable determinant of sedentariness that could be targeted in interventions aiming to reduce sedentary time and improve pertinent health outcomes in RA.

Thomsen et al. [49] delivered a 16 weeks intervention aiming to reduce sedentary time in people with RA. This intervention was based on behavioural choice theory [50] and aimed to “reduce sitting time” via motivational counselling. This intervention was effective at reducing activPAL-assessed sedentary time, and synonymous improvements in health were observed (e.g., pain measured via visual analogue scale). However, whilst demonstrating some success, the psychological determinant targeted (self-efficacy) was not identified based on prior evidence for the role of self-efficacy for influencing sedentary behaviour among people with RA. Moreover, only “general self-efficacy” was assessed, rather than “self-efficacy to reduce sitting time” specifically. This is incongruent with the way the intervention was framed (i.e., with reference to one’s belief about their capability to reduce their sitting time). The present study aimed to overcome this limitation by having the psychological construct of interest be specific to the behaviour—autonomous motivation *to reduce sedentary behaviour*—which provides relatively more scope for identifying autonomous motivation to reduce sedentary behaviour as a potential modifiable intervention target.

The current study demonstrated small to moderate significant associations between quality of motivation to reduce sedentary behaviour, activPAL3^μ^-assessed behaviours and RA health outcomes in bivariate correlation analyses, which were further explored in path analyses in a sequential manner. Interestingly, path analyses revealed change in autonomous motivation to reduce sedentary behaviour was positively associated with change in standing time. In turn, change in standing time was negatively related to change in CRP, pain and fatigue in people with RA. Results also indicated sedentary time and standing time exhibited a strong inverse correlation, which may reflect high shared variance between the two behaviours, rather than associations between sedentary and standing time with RA outcomes being entirely independent of one another. That is, the significant positive association between autonomous motivation to reduce sedentary behaviour with standing time may, to some extent, represent the relationship that autonomous motivation to reduce sedentary behaviour holds with sedentary time, rather than the proposition that people with RA were autonomously motivated to stand per se. The high correlation between sedentary and standing time may suggest that these behaviours are likely to displace each other, which may be why the observed links between sedentary time and standing time with RA outcomes were of similar effect sizes in path models (e.g., sedentary time → CRP, *β* = 0.38; standing time → CRP, *β* = − 0.40). However, experimental studies which aim to target reductions in sedentary time are required to examine the extent to which standing may displace sedentary time in RA, and the subsequent impact on health outcomes in this population.

Results from the current study suggest that encouraging people with RA to try and reduce their sedentary time through standing more, offers a pragmatic health promotion message in this patient group. In addition, our results suggest such an approach may lead to reductions in CRP, pain and fatigue. In line with this, new national PA and sedentary behaviour guidelines have endorsed engagement in LPA (e.g., standing) specifically to replace sedentary time, in adults and older adults [51].

Change in autonomous motivation to reduce sedentary behaviour was positively related to change in stepping time but, in turn, change in stepping time was not associated with any RA outcomes assessed in this study. ActivPAL3^μ^-assessed stepping time is an indicator of total PA. Higher levels of total PA have been demonstrated to reduce the risk of premature all-cause mortality in middle-aged and older adults [52], but it might be that changes in lower-intensity behaviours hold stronger implications for changes in clinically- and patient-important outcomes in RA. Future research should investigate whether total PA or its specific intensities are more important for changes in these RA outcomes.

The present findings are important, when we consider that there is a lack of information regarding the determinants of and health outcomes associated with reducing sedentary time, relative to promoting MVPA, in people with RA [53]. Still, it is important to note that autonomous motivation to reduce sedentary behaviour only accounted for approximately 19%, 14% and 11% of the variance in sedentary, standing and stepping time in the path analyses, respectively. Thus, adopting a more comprehensive approach towards identifying other modifiable determinants of time spent in these behaviours in people with RA at the individual, environmental and organisational level, as well as the inter-relationships between these factors, seems prudent [14].

No significant association between controlled motivation to reduce sedentary behaviour with sedentary, standing or stepping time emerged in this study. The importance of “reducing sedentary behaviour” is a relatively new addition to national guidelines and is a particularly novel area in RA research. People with RA, as well as the people they interact with (e.g., family, friends, clinicians), may be unaware of the adverse health impacts of “too much sitting”. Therefore, these participants may not have experienced any controlled forms of motivation to reduce their sedentary behaviour (e.g., guilt for not reducing their sedentary behaviour or other people telling them to reduce their sedentary behaviour), which may explain the null association between controlled motivation to reduce sedentary behaviour with activPAL3^μ^-assessed behaviours in this study.

Limitations of this study include a small and relatively homogenous sample. To address concerns related to reduced sample sizes, bootstrapping was employed in path analyses to increase statistical power. However, issues related to the composition of hypothesised models remained (e.g., more complex structural equation models could not be tested). In addition, the 6 months period between data collection at T1 and T2 means the season in which data was collected may have influenced movement behaviours among the sample. However, data was collected over a 12 months period, which may attenuate any seasonal influence on the data.

Some selection bias may have been introduced into this study due to its observational design, and focus on movement behaviours (i.e., sedentary behaviour, physical activity) and health. Indeed, it is possible that the participants in this study were more motivated to take part in this research than other individuals in the overall RA population. In addition, participants were mostly female, and had moderate disease activity and functional disability. These sample characteristics limit the ability to generalise current findings to males with RA, those with more/less active disease and functional disability, and those with less interest in their movement behaviours and health. It should be noted, however, that there is a higher prevalence of females in the RA population relative to males [1], and participants’ disease activity (DAS-28) and physical function (HAQ) in this study were similar to findings in previous RA studies [54]. Nevertheless, follow-up research should strive to secure larger samples more representative of males, those with more/less active disease and functional disability, and those less motivated to participate in research in this field.

## Conclusion

This is the first study using SDT-based models of sedentary behaviour change in people with RA. Findings indicate autonomous motivation to reduce sedentary behaviour is predictive of variability in sedentary and standing time among people with RA, to the extent that it may hold implications for clinically- and patient-important health outcomes. As such, results indicate that autonomous motivation to reduce sedentary behaviour might be a viable and malleable target in interventions aiming to attenuate the burden of disease for people with RA, via sedentary behaviour change (e.g., decrease sedentary time, increase standing time).

## Data Availability

The datasets used and/or analysed during the current study are available from the corresponding author on reasonable request.

## Abbreviations

RA: Rheumatoid arthritis
PA: Physical activity
MVPA: Moderate-to-vigorous-intensity physical activity
METs: Metabolic equivalents
LPA: Light-intensity physical activity
SOS-framework: Systems of sedentary behaviours
SDT: Self-determination theory
CRP: C-reactive protein
T1: Time 1
T2: Time 2
HAQ: Health assessment questionnaire
ADLs: Activities of daily living
BREQ-2: Behavioural regulation in exercise questionnaire-2
ESR: Erythrocyte sedimentation rate
DAS-28: Disease activity score-28
MPQ: McGill pain questionnaire
MAF: Multidimensional assessment of fatigue scale
CI: Confidence intervals
CFI: Comparative fit index
TLI: Tucker lewis index
RMSEA: Root mean square error of approximation
DMARDs: Disease-modifying anti-rheumatic drugs
Anti-TNF: Anti-tumour necrosis factor
NSAIDs: Non-steroidal anti-inflammatory drugs
